# Establishment of a Panel of Human Cell Lines to Identify Cellular Receptors Used by *Enteroviruses* to Infect Cells

**DOI:** 10.3390/ijms26030923

**Published:** 2025-01-22

**Authors:** Anastasiia O. Sosnovtseva, Thi Hoa Le, Dmitry S. Karpov, Pavel O. Vorobyev, Yana D. Gumennaya, Olga N. Alekseeva, Peter M. Chumakov, Anastasia V. Lipatova

**Affiliations:** 1 Center for Precision Genome Editing and Genetic Technologies for Biomedicine, Engelhardt Institute of Molecular Biology, Russian Academy of Sciences, 119991 Moscow, Russia; aleom@yandex.ru (D.S.K.); pavel.gealbhain@gmail.com (P.O.V.); chumakovpm@yahoo.com (P.M.C.); lipatovaanv@gmail.com (A.V.L.); 2 Engelhardt Institute of Molecular Biology, Russian Academy of Sciences, 119991 Moscow, Russia; yanagumenny@mail.ru (Y.D.G.); olga_aleks@eimb.ru (O.N.A.); 3 Department of Molecular Microbiology and Immunology, Norris Comprehensive Cancer Center, Keck School of Medicine, University of Southern California, Los Angeles, CA 90033, USA; hoale.kp@gmail.com

**Keywords:** CRISPR/Cas9, *Enteroviruses*, viral phylogeny, coxsackievirus, echovirus

## Abstract

Non-pathogenic natural and recombinant strains of human *Enteroviruses* are the subject of ongoing study with some strains having been approved for use as anticancer agents. The efficacy of oncolytic virotherapy depends upon identifying the receptor utilized by a specific strain for cell entry, and the presence of this receptor on the surface of cancer cells. Accordingly, a rapid and straightforward approach to determining the enteroviral receptors is necessary for developing an effective patient-specific, virus-based cancer therapy. To this end, we created a panel of seven lines with double knockouts on the background of the HEK293T cell line, which lacks the *IFNAR1* gene. In these lines, the main viral receptor genes, including *PVR*, *CXADR*, *CD55*, *ITGA2*, *SCARB2*, *ICAM1*, and *FCGRT*, were knocked out using the CRISPR/Cas9 system. The panel of lines was validated on twelve different *Enteroviruses* types, providing a basis for studying the molecular mechanisms of enterovirus entry into cells, and for developing new therapeutic strains.

## 1. Introduction

*Enteroviruses* are a genus of viruses in the family Picornoviridae. They include positive-sense, single-stranded, non-enveloped RNA viruses with a genome length of about 7500 nt. Enterovirus infections are primarily enteric, and range from asymptomatic and mild flu-like colds to serious illnesses such as meningitis and poliomyelitis [1]. According to modern classifications, viruses belonging to the genus *Enterovirus* are divided into nine distinct species, as follows: *Enterovirus* A, B, C, D, E, F, G, H, and J. Additionally, three rhinovirus species have been identified: *Rhinovirus* A, B, and C [2].

The small genome of *Enteroviruses* encodes regulatory proteins that manipulate intracellular host cell factors to ensure effective reproduction of viral particles. In addition, these viruses rely heavily on host factors for entry. Different species, serotypes, and individual strains of *Enteroviruses* can enter cells using a variety of cell surface proteins that act as receptors for viral attachment and subsequent endocytosis [3,4]. Polioviruses (PV) (*Enterovirus C*) gain entry to cells by binding to the surface protein CD155, a product of the *PVR* gene. This protein forms intercellular adhesion junctions between epithelial cells [5] and some intercellular regulatory interactions within immune cells [6]. Coxsackie B (CVB) and certain echoviruses (E) belonging to *Enterovirus B*, employ the CAR immunoglobulin superfamily protein, a product of the *CXADR* gene [7,8]. Some members of Coxsackie A (CVA), including CVA7, CVA14, CVA16, and Enterovirus 71 (EV-A71) (*Enterovirus A*), utilize the type B2 scavenger receptor (SCARB2) [9], also known as lysosomal integral membrane protein II, LIMP-2v or CD36b-like protein-2; this protein is involved in vesicular transport [10]. E1 (*Enterovirus B)* has been demonstrated to utilize integrin α2β1 (VLA-2; collagen and laminin receptor) [11,12], CVA9 (*Enterovirus B)* utilizes the αVβ and αVβ6 integrins [13,14], CVA21 [15,16] and CVA11 [17] (*Enterovirus C*) utilizes an integrin molecule (ICAM-1), and many members of the echovirus group (*Enterovirus B)* utilize the neonatal Fc receptor FcRn, which consists of two subunits (the *FCGRT* gene product and β2-microglobulin) [18]. In addition to these receptors, some enteroviruses may also bind to the CD55 coreceptor, or DAF, which usually protects cells from the toxic effects of complement [15,19,20]. The binding of the virus to CD55 does not stimulate the uncoating and release of the viral genome; however, it does promote virion attachment to the cell surface, thereby increasing the likelihood of binding to another receptor on the cell surface [21].

The RNA genome of enteroviruses is unstable and subject to frequent mutations and recombinations, which occur predominantly among members of the same species; this determines their evolution [22]. Recombinations and mutations can lead to the emergence of new strains [23] with altered virulence, antigenicity, transmissibility, and drug resistance. In some cases, these are associated with changes in the receptor through which the virus enters the cell [24].

Non-pathogenic and recombinant strains of enteroviruses are being extensively investigated as potential agents for oncolytic virotherapy [25,26] with some strains approved for melanoma treatment in Latvia [27,28]. The high recombination ability of enteroviruses facilitates the creation of improved oncolytic strains with an increased specificity to tumor cells by altering the tropism of the virus [29]. The efficacy of oncolytic therapy is contingent upon identifying the receptor utilized by a specific strain, and the presence of this receptor on the surface of cancer cells [30]. It is therefore imperative to develop a rapid and straightforward method for determining the receptor that enteroviruses use for cellular entry, in order to accelerate the development of an effective patient-specific, virus-based cancer therapy. Another constraining factor regarding the use of oncolytic viruses pertains to pre-existing immunity, a consequence of the pervasive circulation of enteroviruses within the human population. Although there is evidence that pre-existing immunity does not influence the anti-cancer effects of oncolytic viruses, for example oncolytic CVA21 [31], further research is necessary to fully understand the implications of pre-existing immunity on the efficacy of oncolytic viruses. Furthermore, studies have demonstrated that the level and distribution of neutralizing antibodies to certain serotypes of oncolytic enteroviruses is not significant [32]. Nonetheless, an effective approach to the development of personalized medicine entails the preventive determination of neutralizing antibodies to a particular virus strain.

In this study, we established a panel of cells with knockouts of various receptors employed by enteroviruses for cellular invasion, thereby enabling us to ascertain the receptors essential for enterovirus infection of cells.

## 2. Results

### 2.1. Phylogenetic Analysis of Enterovirus Structural Proteins

**Figure 1:**
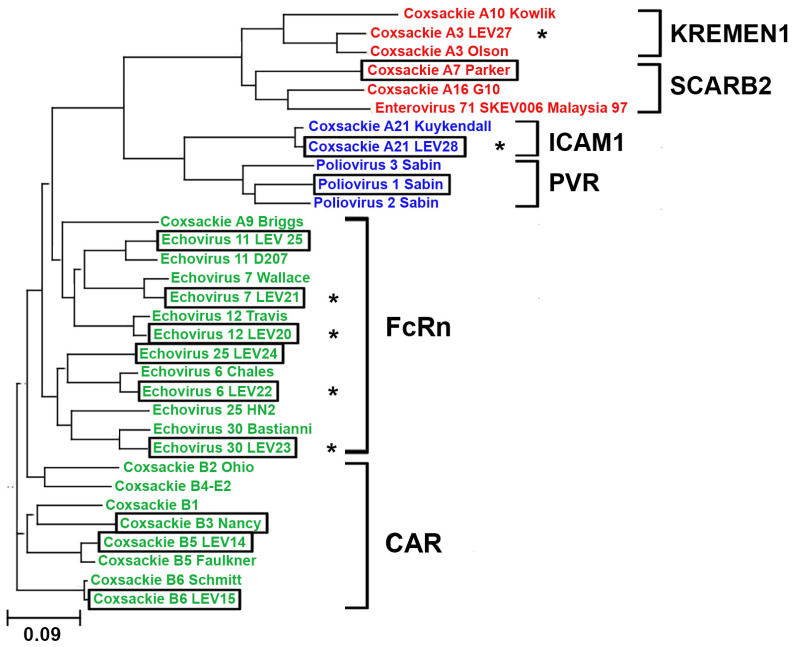
Phylogenetic tree of Enterovirus structural proteins, including the strains used in this work. Strains belonging to groups A, B, and C are colored red, green, and blue, respectively. New strains with sequenced genomes are marked with an asterisk. Strains with names in rectangles were used in this work. The names of the main viral receptors are also indicated.

A phylogenetic analysis was conducted on the structural protein sequences of 32 enterovirus strains. The sequences of 22 strains were obtained from the GeneBank database, and the sequences of 10 were obtained via the laboratory collection of live vaccine enteroviruses, including six new strains whose genomes were sequenced. Notably, the novel strains exhibited high similarity to their respective prototypes, as illustrated in Figure 1. Moreover, the phylogenetic analysis facilitated the prediction of the receptor utilized by the novel viral strain for cellular entry. Our data suggests that the novel strain Coxsackie A21 (LEV28) should interact with ICAM1, and the echovirus strains 7 (LEV21), 12 (LEV20), 6 (LEV22), and 30 (LEV23) should enter cells via an interaction with FCGRT. The construction of the phylogenetic tree did not allow for the identification of viruses that utilize CD55 to infect cells within a discrete cluster. This may be attributed to the role of CD55 in cell infection, as it only facilitates virion attachment to the cell surface [21].

### 2.2. Obtaining a Panel of Cell Lines with Knockouts of Different Enterovirus Receptors

The results of the phylogenetic analysis needed an experimental verification. Consequently, we devised an experimental approach for the validation of the viral receptors. The experimental approach is based on testing the reproduction of enteroviruses in seven HEK293 lines carrying knockouts of known enteroviral receptors.

To obtain the knockout lines, the CRISPR/Cas9 system was employed to target genes encoding seven surface molecules (PVR, CAR, CD55, SCARB2, VLA-2, ICAM1, and FcRn) for which a role as an enteroviral receptor or coreceptor has been described. Each gene knockout was introduced into the HEK293TΔIFNAR1 cell line, previously generated by knockout of the *IFNAR1* gene encoding the type I interferon receptor subunit [33]. Due to its impaired cellular interferon response, this cell line can support the replication of many enteroviruses, including non-pathogenic ones.

**Figure 2:**
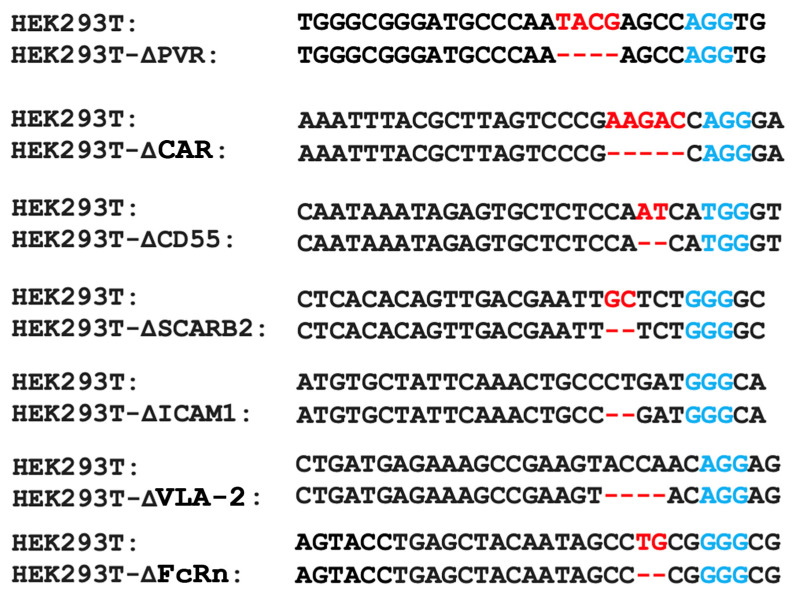
Sequences of enterovirus receptor gene regions in the CRISPR/Cas9-induced knockout line panel. Blue letters are PAMs. Red letters and dashes are changes in gene sequences introduced by the CRISPR/Cas9 system.

The cells were transfected with the pCas-Guide-P2A-RFP plasmid, which contained inserted sites encoding gRNA spacers targeting the corresponding gene. Following transfection, monoclonal cultures were obtained and subsequently analyzed by sequencing the Cas9-targeted gene region. The most promising clones were then selected based on the results of this sequencing. Consequently, a panel of seven HEK293T cell lines with two gene knockouts (*IFNAR1*, and one of the genes encoding receptor proteins for enteroviruses) were obtained (Figure 2). The knockout cell lines were tested by western blotting analysis, which confirmed the absence of the proteins of interest.

### 2.3. Evaluation of Enterovirus Production in a Panel of Cells with Viral Receptor Knockouts

**Figure 3:**
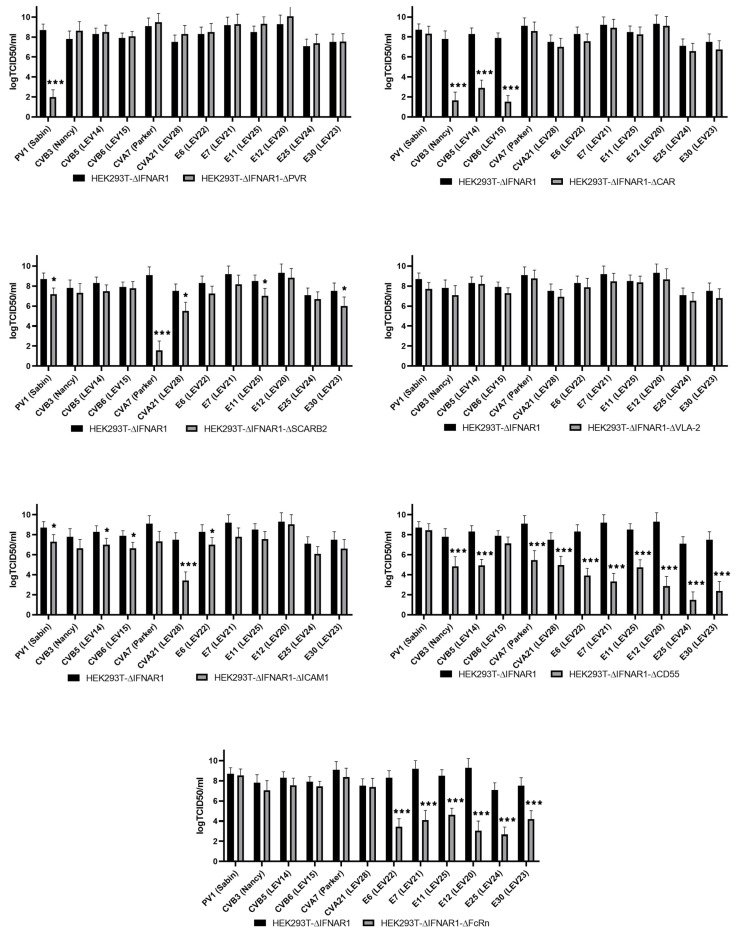
Level of enterovirus titers on cell lines with the knockout of genes encoding the main enterovirus receptors. Results from three independent experiments are presented. Error bars represent standard deviation. Statistical significance: * *p*-value between 0.05 and 0.01; *** *p*-value < 0.001, according to Student’s *t*-test.

This study aimed to assess the production rate of 12 different enteroviruses on the HEK293T-ΔIFNAR1 cell line and its derivatives, with an additional knockout of one of the viral receptors. The results of this assessment are presented in Figure 3.

As observed, the knockout of PVR resulted in a notable reduction in PV1 (Sabin) production. The replication of all the strains remained unimpaired, this being consistent with the data on the receptor properties of PVR [34].

The inactivation of *CXADR* gene expression resulted in a significant reduction of viral titer for only coxsackieviruses of group B (CVB3 (Nancy), CVB5 (LEV14), and CVB6 (LEV15)). This finding is consistent with the available data on the role of the CAR molecule in the infection of cells with some representatives of coxsackieviruses of group B [35]. Previously, comparable data were obtained for CVB5 (LEV14) and CVB6 (LEV15) on the HEK293T cell line with CAR knockout [36]. 

The inactivation of the *ICAM1* gene has been demonstrated to significantly reduce the replication of CVA21 (LEV28), a finding that is consistent with the existing literature [15]. We also note that a slight effect was observed for PV1 (Sabin), CVB5 (LEV14), CVB6 (LEV15), and E6 (LEV22).

The knockout of the *SCARB2* gene suppressed the production of only CVA7 (Parker), which is consistent with previously published data [9]. Nevertheless, a slight impact was observed in the level of PV1 (Sabin), CVA21 (LEV28), E11 (LEV25), and E30 (LEV23) production.

The knockout of *ITGA2* did not result in a reduction in production of any of the strains under study, which is also consistent with previous research findings.

The inactivation of the *CD55* gene compromised the production rate of numerous enteroviruses, albeit to varying degrees. The replication of CVB3 (Nancy) and CVB5 (LEV14) viruses was reduced by three orders of magnitude. In comparison, the production rate of CVA7 (Parker) and CVA21 (LEV28) viruses was decreased by two and a half to three orders of magnitude, and that of E6 (LEV22), E7 (LEV21), E11 (LEV25), E12 (LEV20), E25 (LEV24), and E30 (LEV23) was reduced by four orders of magnitude. The replication of E25 (LEV24) was notably diminished, exhibiting a reduction of up to seven orders of magnitude. The broad impact of CD55 on the infection of cells by a range of enterovirus strains has been previously documented, including CVA21 [15], CVB3, and CVB5 [15], as well as E6 [19], E7 [15], E11 [37], E12 [38], and E30 [39]. The observed effect of CD55 on cell infection with CVA7 or E25 strains has not been previously reported. Furthermore, our findings indicate that CD55 is not a requisite factor for infecting cells with PV1 (Sabin) and CVB6 (LEV15).

The knockout of *FCGRT* resulted in the complete cessation of production of E6 (LEV22), E7 (LEV21), E11 (LEV25), E12 (LEV20), E25 (LEV24), and E30 (LEV23), while no effect was observed on the level of coxsackie and polioviruses production. This finding is consistent with the existing literature on the role of the FcRn in the infection of cells by certain representatives of the species *Enteroviruses B* [18].

## 3. Discussion

The resulting panel of cells serves as a convenient tool for determining the viral receptor utilized by a specific enterovirus strain for cell entry. Upon verification of the obtained cell panel, the majority of the enterovirus strains studied exhibited results consistent with the phylogenetic analysis and previously obtained data from other researchers in the field. 

The availability of experimental data indicating whether viruses use or do not use specific receptors may prove invaluable in the development of oncolytic therapy based on enteroviruses. Due to the inherent instability of the enterovirus genome and the potential for accumulation of mutations, the resulting panel of viruses can be employed to generate viruses that utilize alternative receptor molecules to infect cells through bioselection. For example, the evidence suggests that FCGRT may play a role in the antitumor immune recognition of tumor cells at an early stage of tumor growth [40]. Additionally, a decrease in FcRn expression has been observed to correlate with tumor progression [41]. Consequently, the search for, and selection of enteroviruses with FcRn-independent penetration mechanisms can be achieved using this panel.

In addition, the different levels of suppression of viral reproduction in the knockout cell lines may indicate the degree of dependence of a given viral strain on the corresponding receptor for cell entry. We hypothesize that the stronger the level of reproduction suppression of a viral strain in a given knockout line, the stronger the dependence of that strain on a given receptor. This feature of our panel can be used for a preliminary analysis of mutant viral strains with altered receptor affinity, which can then be validated by more precise methods.

Furthermore, the identification of the receptor required for infection of tumor cells with a specific strain can be used for the selection of the most effective treatment on a case-by-case basis. This procedure can be performed at the stage of histological examination of patient tumor samples by various methods (immunocytochemistry, transcriptome analysis for enterovirus receptors, etc.). In conjunction with the assessment of serum neutralizing antibody levels, this approach can markedly enhance the efficacy of oncolytic virus therapy. Consequently, the constructed cell panel may prove advantageous for the isolation of enteroviruses exhibiting a heightened oncolytic potency. This may be achieved by identifying more appropriate viruses according to the expression levels of different enterovirus receptors, and further delineating genome alterations that result in modifications to the cell penetration pathway. This can then be employed to generate recombinant strains with defined characteristics.

Thus, the use of oncolytic enteroviruses could be an effective approach to antitumor therapy, especially within the framework of personalized medicine. Such an approach would not only allow for the selection of specific strains, but also the use of successful strains. This would avoid the emergence of recurrent resistance in the face of the development of an antiviral immune response. The safety of an enterovirus-based therapy is attributed to the use of enterovirus strains of limited pathogenic potential and the availability of antiviral drugs such as ribavirin, which has a demonstrated efficacy against enteroviruses [42,43].

## 4. Materials and Methods

### 4.1. Phylogenetic Analysis of the Enteroviruses

The full-genome nucleotide sequences of the available enterovirus strains were retrieved from the GenBank database. The genomes of the novel strains were sequenced using the Illumina platform. The sequences of the structural proteins were obtained and used in a multiple protein sequence alignment, followed by the construction of a phylogenetic tree, which was performed using Clustal Omega [44] on the EMBL-EBI site (https://www.ebi.ac.uk/ accessed on 21 November 2024). Visualization of the phylogenetic tree was performed in the Phylogenetic tree (newick) viewer [45] (http://etetoolkit.org/treeview/ accessed on 21 November 2024).

### 4.2. Cell Lines and Viral Strains

The human embryonal rhabdomyosarcoma (RD) (CCL-136) and HEK293T (CRL-3216) cell lines were procured from the ATCC bank. The IFNAR1-knockout HEK293T cell line was previously established [33]. The cells were cultured in a DMEM medium containing 100 μg/mL penicillin/streptomycin, and 10% fetal bovine serum in an atmosphere of 5% CO_2_ at 37 °C.

The Sabin vaccine strain of poliovirus type 1 (PV1 (Sabin)); Echovirus type 6, LEV22 (E6 (LEV22)); Echovirus type 7, strain LEV21 (E7 (LEV21)); Echovirus type 11, strain LEV25 (E11 (LEV25)); Echovirus type 12, strain LEV20 (E12 (LEV20)); Echovirus type 25, strain LEV24 (E25 (LEV24)); Echovirus type 30, strain LEV23 (E30 (LEV23)); Coxsackie virus A7, strain Perker (CVA7 (Parker)), Coxsackie A21 virus, LEV28 (CVA21 (LEV28)); Coxsackie B3 virus, strain Nancy (CVB3 (Nancy)), Coxsackie B5 virus, strain LEV14 (CVB5 (LEV14)), and Coxsackie B6 virus, strain LEV15 (CVB6 (LEV15)) were obtained from a laboratory collection. The enteroviruses were amplified on the RD cell line. The infected cells and culture supernatant were collected within 2–3 days, after which the cells were destroyed by three cycles of freezing, and purified by centrifugation at 2000× *g* for 10 min. Virus titers were determined using the standard Reed-Muench method.

### 4.3. The HEK293T Cell Lines with Viral Receptor Gene Knockouts

Based on the CRISPR/Cas9 technology, the knockout system generated the lines of HEK293TΔIFNAR1 cells, with an additional knockout of genes encoding the receptor proteins for enteroviruses. To this end, plasmid pCas-Guide (Origene, Rockville, MD, USA) was modified by the insertion of a gene encoding red fluorescent protein RFP as the second expressed translation frame, which was placed after the Cas9 gene via element 2A, thereby facilitating polycistronic translation. The pCasGuide-2A-RFP plasmid constructs, which express sgRNA spacers specific to enterovirus receptor gene sites, were obtained by cloning the oligonucleotides from Table 1, as previously described [33]. Subsequently, the cells were transfected with calcium phosphate in accordance with a previously described methodology [46]. Cell clones were subsequently obtained as previously described [33]. Briefly, the genomic DNA of cell clones was isolated using a specialized ExtractDNA Blood & Cells kit (Evrogen, Moscow, Russia) using the manufacturer’s instructions. The CRISPR/Cas9-mediated editing of genes encoding the viral receptors was confirmed by PCR with the primers indicated in Table 1, followed by Sanger sequencing.

### 4.4. Enterovirus RNA Purification and Genome Sequencing

Novel strains of enteroviruses were produced in the RD cell line. The harvested medium was clarified from the cell debris by centrifugation at 4 °C, and 2500 rpm for 15 min. The virus-containing supernatant was stored in 0.5 mL aliquots at –80 °C. The viral RNA was extracted using the GeneJET Viral DNA/RNA Purification Kit (Thermo Fisher Scientific, Waltham, MA, USA) using the manufacturer’s instructions. The quality and quantity of the RNA was evaluated through spectrophotometry and RNA gel electrophoresis. The cDNA synthesis was conducted using Mint reverse transcriptase (Evrogene, Moscow, Russia) with N6 random primers, in accordance with the manufacturer’s instructions. The enterovirus’s cDNA was used to prepare the sequencing library with a Nextera DNA Flex Library Preparation Kit (Illumina, San Diego, CA, USA), according to the manufacturer’s protocol. The size of the genomic library was analyzed on an Agilent 2100 Bioanalyzer (Thermo Fisher Scientific, Waltham, MA, USA) being about 500 bp. The library was sequenced on the Illumina MiSeq System using the MiSeq Reagent Micro Kit v2 (300 cycles). The draft enteroviral genomes were assembled de novo using a SPAdes assembler [47] on the Galaxy web service [48] with automatic k-measure size selection; other parameters were set as default. The serotype identification of the viruses from the laboratory collection were determined by subjecting the amino acid sequence of the P1 regions of the genome to a blast against the virus genomes in the GeneBank database (Table 2).

### 4.5. Determination of Enterovirus Production

To determine the production rate, the double-knockout and control cell lines, HEK293TdIFNAR1, were infected with enteroviruses at an MOI of 0.001. After three days, the medium containing the viruses was harvested, cleared by centrifugation for five minutes at 2000 g, and used to determine the titer of the virus using the Reed-Muench method. To this end, the RD cells were infected with 10-fold dilutions of the collected medium, and the titer was calculated after three days. The experimental procedure was replicated three times, with each iteration comprising four technical replicates.

## Figures and Tables

**Table 1:** The sequences of sgRNA spacers, the primers for PCR amplification, and the antibodies used to generate and validate the enterovirus receptor gene knockout.

Viral Receptor Target Gene	Oligonucleotides Used for Cloning of sgRNA Spacers	Oligonucleotides Used for Amplification of CRISPR/Cas9 Targeted Gene Regions	An Antibody Used Against a Viral Receptor
*PVR*	5′-phospho-GATCGcgggatgcccaatacgagccG	CCATGCCATCCTGTACCCTT	Abcam (ab205304)
	5′-phospho-AAAACggctcgtattgggcatcccgC	GAAGCAATGCCTACAGTGCC	
*CXADR*	5′-phospho-GATCGtacgcttagtcccgaagaccG	TTCTGAATGGCTGCGGGG	Abcam (ab126250)
	5′-phospho-AAAACggtcttcgggactaagcgtaC	ATGTGACTGGCAAGGTGATG	
*CD55*	5′-phospho-GATCGattggagagcactctattG	CCAGCACCACCACAAATTGA	Abcam (ab253284)
	5′-phospho-AAAACaatagagtgctctccaatC	AGACCCTTCTGAGAAGCTGT	
*SCARB2*	5′-phospho-GATCGacagttgacgaattgctctggG	ATCGAGGCCATGTTGAAAGC	Abcam (ab176317)
	5′-phospho-AAAACccagagcaattcgtcaactgtC	GTTAATCTGGCTTGGGGTGC	
*ICAM1*	5′-phospho-GATCGgctattcaaactgccctgatgG	GCGCACATTCCCCTTGATGAA	Abcam (ab109361)
	5′-phospho-AAAACcatcagggcagtttgaatagcC	CAGTACACGGTGAGGAAGGT	
*ITGA2*	5′-phospho-GATCGattggagagcactctattG	TGTAGCCACAAGACACTGATG	Abcam (ab181548)
	5′-phospho-AAAACgttggtacttcggctttctcC	ACAGCAAAAGGATTCCAGCA	
*FCGRT*	5′-phospho-GATCGctgagctacaatagcctgcgC	TTCTCCCTCCCTGGGTATCT	Abcam (ab228975)
	5′-phospho-AAAACaccggaatctcaccttttcccG	ACCGGAATCTCACCTTTTCCC	

**Table 2:** Identification of serotypes of the laboratory viruses used in the work.

Name of Strain	Identified Virus	Strain from Gene Bank	Percent Identity
*LEV14*	*CVB5*	SPS68033 (CVB5 strain RO-14-5-70)	97.27%
		AF114383 (CVB5 strain Faulkner)	96.02%
*LEV15*	*CVB6*	AFD32988.1 (CVB6 strain LEV15)	100%
		AAF12719 (CVB6 strain Schmitt)	99.18%
*LEV20*	*E12*	AWX63811 (E12 strain K624/YN/CHN/2013)	97.63%
		Q66575 (E12 strain Travis)	96.17%
*LEV21*	*E7*	AZT88545 (E7 strain Nigeria/AFP/2014)	97.53%
		AY302559 (E7 strain Wallace)	94.2%
*LEV22*	*E6*	AXP11726 (E6 strain GHA:CEN:UDW/2017)	99.10%
		Q66474 (E6 strain Charles)	95%
*LEV23*	*E30*	WGU13597 (E30 strain E30/USA/3E5/2009)	93.69%
		AF311938 (E30 strain Bastianni)	92.45%
*LEV24*	*E25*	CAA62257 (E25 strain JV-4)	98.13%
		ADG63656 (E25 strain HN2)	95.74%
*LEV25*	*E11*	QIZ12960 (E11 strain SD2003-478/SD/CHN/2003)	97.53%
		ABV00677 (E11 strain D207)	91.80%
*LEV28*	*CVA21*	ABM54535 (CVA21 strain USA-Az94-10621)	99.77%
		AAQ04838 (CVA21 strain Kuykendall)	97.69%

## Data Availability

The datasets generated during and/or analyzed during the current study are available from the corresponding author upon reasonable request.
